# Monthly Summary

**Published:** 1870-08

**Authors:** 


### MONTHLY SUMMARY.

*Dyspepsia as a Cause of Consumption.*—It is a fact not generally known that dyspepsia is often followed by consumption, and that too, on well established physiological principles. Indeed, it hardly admits of question whether this is not the most fruitful source of phthisis, at the present day. Indigestion begets inanition, and this is but an incipient stage of the disease. A dislike to fatty articles of diet is the most marked symptoms of this want of assimilation of this much-needed respiratory element. So marked is this physiological fact, that it might be safely predicated of a family in which one child distinguished itself from the others by its dislike of fats, that it will be more prone to become the victim of consumption than any of the others.

The dyspeptic may be known by his frequent complaints of "biliousness," acid eructions after taking food, belching of wind, flatulence, heartburn, &c. Everything of a fatty nature seems to disagree with him, "raising acid," to use a popular phrase; sweetmeats, sugar, coffee, &c., causing uneasines and a sense of weight in the stomah, owing to a want of power to digest them. Then follows emaciation, a disappearance of the adipose tissue by reabsorption into the circulating fluids of the body—for so long as the fires of life burn, they will consume the needed fuel. No readier method could be devised to cause consumption of lung substance, than to refuse to supply them with the carbon derived from fatty food; for the lungs as surely consume carbon as the locomotive engine. To withhold this is but to burn the incense so low as to consume the censer also.

The majority of cases of tuberculosis, whether hereditary or not, are preceded by well-marked dyspeptic symptoms. Indeed there is no reason why it may not be caused *de novo* by these derangements of the digestive organs, for the reasons stated above.

After the adipose tissue stored away in more healthful periods of life has been consumed, there comes a wasting of other tissues of the body, the muscles become flabby and shrink away, the skin becomes thin and transparent, showing the blue tracery of the vein beneath; at last, the lungs, robbed of fuel, burn out as truly oxydized as the fallen tree, over which the green moss has crept for years.—*Oregon Medical & Surgical Reporter.*

---

*Prevention of Iron Rust.*—Dr. Calvert, a fortnight ago, communicated to the Chemical Society some very useful information on the rusting of iron. Rust is mainly sesquioxide of iron, and it has always been supposed that the active agents in producing it are moisture and oxygen. It seems however, from Dr. Calvert's experiments, that carbonic acid must be associated with these to produce any considerablc amount of oxidation. In dry oxygen, iron does not rust at all; in moist oxygen, but little and seldom; but in a mixture of moist carbonic acid and oxygen, iron and steel rust very rapidly. In like manner a piece of bright iron placed in water saturated with oxygen rusts very little; but if carbonic acid is present as well, oxidation goes on so fast that a dark precipitate is produced in a very short time. Curiously enough, bright iron placed in a solution of caustic or carbonated alkali does not rust at all. These facts show that the points to be attended to in the preservation of iron from rust are the exclusion of carbonic acid and moisture, two indications which may be very easily fulfilled.—*Druggist's Circular.*

---

*The Weight of the Brain.*—In the *Journal of Mental Science*, 1866, there is an elaborate paper by Dr. Shuman on the "Weight of the Human Brain," and he gives the following interesting table of the brain-weight of fifteen distinguished men.—

| | Age. | Oz. |
|---|---|---|
| 1. Cuvier, naturalist | 63 | 64·5 |
| 2. Abercrombie, Physician | 64 | 63 |
| 3. Spurzheim, Physician | 56 | 55·06 |
| 4. Dirichlet, mathematician | 54 | 53·6 |
| 5. De Morny, statesman and courtier | 50 | 53·6 |
| 6. Daniel Webster, statesman | 70 | 53·5 |
| 7. Campbell, Lord Chancellor | 80 | 53·5 |
| 8. Chalmers, celebrated preacher | 67 | 53 |
| 9. Fuchs, pathologist | 52 | 52·9 |
| 10. Gauss, mathematician | 78 | 52·6 |
| 11. Dupuytren, Surgeon | 58 | 50·7 |
| 12. Whewell, philosopher | 71 | 49 |
| 13. Hermann, philologist | 51 | 47·9 |
| 14. Tiedemann, physiologist | 80 | 44·2 |
| 15. Hausmann, mineralogist | 77 | 43·2 |
| Averages of ten distinguished men | 50-70 | 54·7 |
| Averages of fifteen distinguished men | 50-80 | 52·7 |

The average brain weight, between the ages of 20 and 60, of Scotchmen is given by Dr. Peacock at 50 ounces, and from these figures it is evident that whilst Sir James Simpson's brain is much above the average of his countrymen, it is really lower than that of the brains of the ten distiuguished men given in the preceding table who died between 50 and 70.

*Chloral Hydrate.*—This interesting drug has now been sufficiently tested by a large number of eminent practitioners to enable us to form a tolerably clear idea both of its merits and defects; and as we percieve that (after the usual fashion in matters medical) there is going to be an epidimic rage for the new remedy, it may be well to call the attention of the public to the principle features of its action which can be said to be fairly made out.

In the first place, the term "anæsthetic" should no longer be applied to chloral, for it has entirely failed to make good its claim to this reputation; even the largest doses do but produce a heavy and prolonged sleep, which is, however, essentially different from true anæsthesia. On the other hand, as a producer of sleep, chloral is, in many respects, unrivalled: for though, like every other remedy, it fails in a considerable number of cases, it does succeed in a very large number; in fact, it is inferior in certainty, as a hypnotic, to opium, and almost every other drug, in the charater of its sleep producing action; there are no attendant symptoms of cerebral oppression; the sleep, though often prolonged, is light and refreshing, and no unpleasant after-symptons are experienced. It is important to observe however, that this description only applies to the use of moderate quantities, and that not only unpleasant, but dangerous symptoms have been produced by doses which we regret to see are very commonly used. Very careful inquiry leads us to assert that it is both unnecessary and dangerous to give larger doses than twenty to thirty grains repeated once or twice if necessary, for hypnotic purposes. Doubtless it might happen that one hundred consecutive patients might take much larger doses with impunity, but the one hundred and first might present the alarming symptoms described by Dr. Reynolds in a recent number of the *Practitioner*, as produced by a dose of fifty grains, and these symptoms might easily take a fatal turn.

As a remedy for pain, chloral holds a very varying place in the estimation of medical men, some rating it highly, and others thinking it almost worthless. Perhaps the safest estimate of its power over pain is that it only exerts an indirect influence, by inducing a disposition to sleep, in which the pain is forgotten. Certainly it has entirely failed, in the hands of the present writer, to relieve pain of a pure neuralgic type. On the other hand, there is a great deal of evidence that it relieves suffering where the parts are very *tense*, and where mere arterial *throbbing* counts for much in the production of the pain ; thus it has been very favorably spoken of for its effects in gout. And this fact, if it be correct, corresponds with certain observations which have been made as to its action on the circulation. Both from sphygmographic experiments on healthy persons and on patients, and also from the details of the nearly fatal case reported by Dr. Reynolds, there is reason to that chloral exerts a contracting influence upon the arterioles, powerful in proportion to the dose ; and it may well be that arterial throbbing is checked by this kind of influence.

On the whole, however, there can be little doubt that the great function of chloral is that of a hypnotic, and calmer of general nervous irritability. In delirium tremens it is excellent; and it is probable that with two such weapons for choice as bromide of potassium and chloral we shall be able almost entirely to dispense with the use of opium, which is so uncertain and dangerous a remedy in that disease. In the state of sleeplessness which threatens the access of peurperal mania, chloral is probably an unequalled remedy. In melancholia, its action as an hypnotic appears to be powerful and remarkably sure. In mania, also, it acts well enough as an hypnotic, though there seems some division of opinion as to whether it does permanent good. We may also state that in the irritable condition of aged persons, who find it difficult to sleep for any lenght of time continuously, the use of a single dose of thirty grains of chloral appears often to answer excellently well. The minor uses of the drug in relieving more trivial conditions of nervous irritation, and in alleviating painful spasmodic symptoms of various kinds, are probably considerable.—*Lancet.*

---

*Inebriation Hereditary.*—Dr. Turner, in his "Second Annual Report of the State Inebriate Asylum," states that out of 1406 cases of delirium tremens which have come under his observation, 980 had an inebriate parent or grandparent,or both. He believes if the history of each patient's ancestors were known, it would be found that 8 out of 10 of them were free users of alcoholic drinks. —*Med. Record.*

*Somnambulism.*—The Archbishop of Bordeaux thus describes a case of somnambulism in a young priest:—He was in the habit of writing sermons when asleep, and although a card was placed between his eyes and the note book, he continued to write vigorously. After he had written a page requiring correction, a piece of blank paper of the exact size was substituted for his own manuscript, and on that he made the corrections in the precise situation they would have occupied on the original page. A very astonishing part of this is that which relates to his writing music in his sleeping state, which it is said he did with perfect precision. He askəd for certain things, and saw and heard such things as bore directly upon the subject of his thoughts. He detected the deceit when water was given to him in the place of brandy which he asked for. Finally he knew nothing of all that had transpired when he awoke, but in his next paroxysm he rememembered all accurately—and so lived a sort of double life, a phenomenon which is said to be universal in all the cases of exalted somnambulism.—*Scientific Press.*

---

*Iodine Gargle.*—M. Cullier (*Union Medicale*) advocates the following in syphilitic ulceration of the mouth and throat, and in œzena: Iodide potassium 1 part, honey syrup 30, and decoction of barley 120 parts.—*Journal of Materia Medica.*

---

